# Detecting the borders between coding and non-coding DNA regions in prokaryotes based on recursive segmentation and nucleotide doublets statistics

**DOI:** 10.1186/1471-2164-13-S8-S19

**Published:** 2012-12-17

**Authors:** Suping Deng, Yixiang Shi, Liyun Yuan, Yixue Li, Guohui Ding

**Affiliations:** 1 School of Life Sciences and Technology, Tongji University, 1239 Siping Road, Shanghai, 200092, P.R. China; 2 Key lab of systems biology, Shanghai Institutes for Biological Sciences, Chinese Academy of Sciences, 320 Yueyang Road, Shanghai, 200031, P.R.China; 3 Shanghai Center for Bioinformation Technology, 100 Qinzhou Road, Shanghai, 200235, P. R. China; 4 Shanghai Jiaotong University, Shanghai, 200240, P. R. China

## Abstract

**Background:**

Detecting the borders between coding and non-coding regions is an essential step in the genome annotation. And information entropy measures are useful for describing the signals in genome sequence. However, the accuracies of previous methods of finding borders based on entropy segmentation method still need to be improved.

**Methods:**

In this study, we first applied a new recursive entropic segmentation method on DNA sequences to get preliminary significant cuts. A 22-symbol alphabet is used to capture the differential composition of nucleotide doublets and stop codon patterns along three phases in both DNA strands. This process requires no prior training datasets.

**Results:**

Comparing with the previous segmentation methods, the experimental results on three bacteria genomes, *Rickettsia prowazekii, Borrelia burgdorferi *and *E.coli*, show that our approach improves the accuracy for finding the borders between coding and non-coding regions in DNA sequences.

**Conclusions:**

This paper presents a new segmentation method in prokaryotes based on Jensen-Rényi divergence with a 22-symbol alphabet. For three bacteria genomes, comparing to A12_JR method, our method raised the accuracy of finding the borders between protein coding and non-coding regions in DNA sequences.

## Background

The prediction of protein coding regions in DNA sequences is a major goal and a long-lasting topic in molecular biology, especially for the genome projects [1-6]. Lots of methods for finding probable borders are based on strong signals between the coding regions and the non-coding ones [7,8]. Staden [9] used the intersection method to detect the borders between coding and non-coding regions. The information entropy measures for signals are useful for identifying the homogeneous regions and evaluating the genomic complexity [10-12]. The entropy-based segmentation methods can be used to identify the borders between coding and non-coding regions [10,13,14]. The Jensen-Shannon divergence measure has provided an impelling tool in doing this [8,9,15]. Bernaola-Galvan et al. presented an entropic segmentation method to search the borders [12]. The accuracy of their results was higher than those obtained with the intersection method [9,12]. The segmentation method presented by Nicorici et al. [8] was based on the Jensen-Rényi divergence measure in both DNA strands. In 2007, Zhang et al. [16] introduced a segmentation method based on a R14 alphabet and the *β-KL *divergence. However, its accuracy is not higher than Nicorici's method [8].

In this study, we constructed a 22-symbol alphabet to represent DNA sequences. Based on the entropy theory, we used recursive segmentation to detect the borders between coding and non-coding DNA regions. Comparing to previous methods, it is shown that our accuracy was well improved.

## Materials and methods

### The data set

Three tested genomes were downloaded from the National Center for Biotechnology Information (https://www.ncbi.nlm.nih.gov/): *Rickettsia prowazekii *(GenBank: AJ235269), *Borrelia burgdorferi *(GenBank: NC_000948 and AE000783) and *E.coli *(GenBank: NC_009837, NC_008563 and NC_010468).

### A22 alphabet

The statistical properties of DNA sequences were commonly used to recognize protein coding regions [11,12,14,17-20]. The statistical properties of doublets of nucleotides (called di-nucleotide for short) in coding regions are also different from those in non-coding regions. This may be used to predict the coding DNA regions. Each di-nucleotide of the DNA sequence is substituted by the symbols from A16 = {A_A_, A_C_, A_G_, A_T_, C_A_, C_C_, C_G_, C_T_, G_A_, G_C_, G_G_, G_T_, T_A_, T_C_, T_G_, T_T_} (Table 1).

**Table 1:** Di-nucleotides mapping in 22-symbol alphabet.

Di-nucleotide	Symbol	Di-nucleotide	Symbol
AA	A_A_	GA	G_A_
AC	A_C_	GC	G_C_
AG	A_G_	GG	G_G_
AT	A_T_	GT	G_T_
CA	C_A_	TA	T_A_
CC	C_C_	TC	T_C_
CG	C_G_	TG	T_G_
CT	C_T_	TT	T_T_

The distribution of stop codon patterns (SCPs for short) in DNA coding regions differs from that in the non-coding regions [3,21]. It is well known that the SCPs are strong signals in DNA sequences, so that we can effectively use these signals to detect borders between coding and non-coding DNA regions [22]. The SCPs, TGA, TAG and TAA appear in one given DNA strand, and the three SCPs corresponding to TCA, CTA and TTA appear on the reverse strand. In this way, the SCPs statistics on both DNA strands is the same as the statistics of the six codons TAA, TAG, TGA, TCA, CTA, and TTA on a single DNA strand.

In our study, we introduced a 22-symbol alphabet (called A22 for short) that took into account the non-uniform distribution of di-nucleotides and SCPs in both DNA strands (Table 1 and Table 2). Thus the di-nucleotides and the SCPs are substituted by the symbols from A22 = {A_A_, A_C_, A_G_, A_T_, C_A_, C_C_, C_G_, C_T_, G_A_, G_C_, G_G_, G_T_, T_A_, T_C_, T_G_, T_T_, S_1_, S_2_, S_3_, S'_1_, S'_2_, S'_3_}(Table 1 and Table 2). The phase of the nucleotide is defined as m = ((n-1)mod 3)+1, where m ∈{1,2,3}, and n is the position of the nucleotide in the DNA sequence. The phase of a SCP is defined in the same way with the exception that n represents the position of the first nucleotide of the given codon. For example, the DNA sequence ACGTAATC is converted using the A22 alphabet as A_C_, C_G_, G_T_, T_A_, S_1_, A_A_, S'_2_, A_T_, T_C_.

**Table 2:** SCPs mapping in 22-symbol alphabet.

Codons	Phase	Symbol
	1	**S_1_**
**TGA, TAG, or TAA**	2	**S_2_**
	3	**S_3_**

	1	**S'_1_**
**TCA, CTA, or TTA**	2	**S'_2_**
	3	**S'_3_**

### Detecting borders between coding and non-coding DNA regions

In order to partition a DNA sequence, we used the approach proposed by Nicorici et al [8]. and Li [10,13]. A sliding pointer is moving along the sequence. At each position, the pointer divided the sequence into two subsequences and we computed the Jensen-Rényi divergence $D_{JR_{a}}$. Then, we found the maximum $D_{JR_{a}}$ and computed its segmentation strength *s *(see below). If this segmentation strength *s *exceeded a given threshold *s*_0_, the position was identified as a significant cut (or a probable border) between coding and non-coding DNA regions. The procedure continued recursively for each of the two resulting subsequences created by each cut until none of the cuts had a segmentation strength level exceeding the *s*_0_. Then such a sequence was segmented at the segmentation strength level *s*_0_.

In this study, the Jensen-Rényi divergence [23,24] is defined as follows:

(1) $$D_{JR_{a}}=\underset{i}{\max}D_{JR_{a}}\left(i\right)=\left[R_{a}-\frac{i}{N}R_{a,l}-\frac{N-i}{N}R_{a,r}\right]$$

Where *R_a_, R_a, l _*and *R_a, r _*are the Jensen-Rényi entropies of the whole, left, and right subsequences, respectively.

### Stopping criterion

To decide when the segmentation process has to be stopped, we adopted the method proposed in references [8,25,26] and introduced a segmentation strength, derived empirically, as

(2) $$s=\frac{2\cdot N\cdot D_{JR_{a}}-K\cdot {log}_{2}N}{K\cdot {log}_{2}N}$$

The recursive segmentation continues as long as *s *≥ *s*_0 _and the segmented sequence has SCPs in all three phases, where *s*_0 _can be set by the user. K is a constant, which was set as 16 [8].

**Figure 1:**
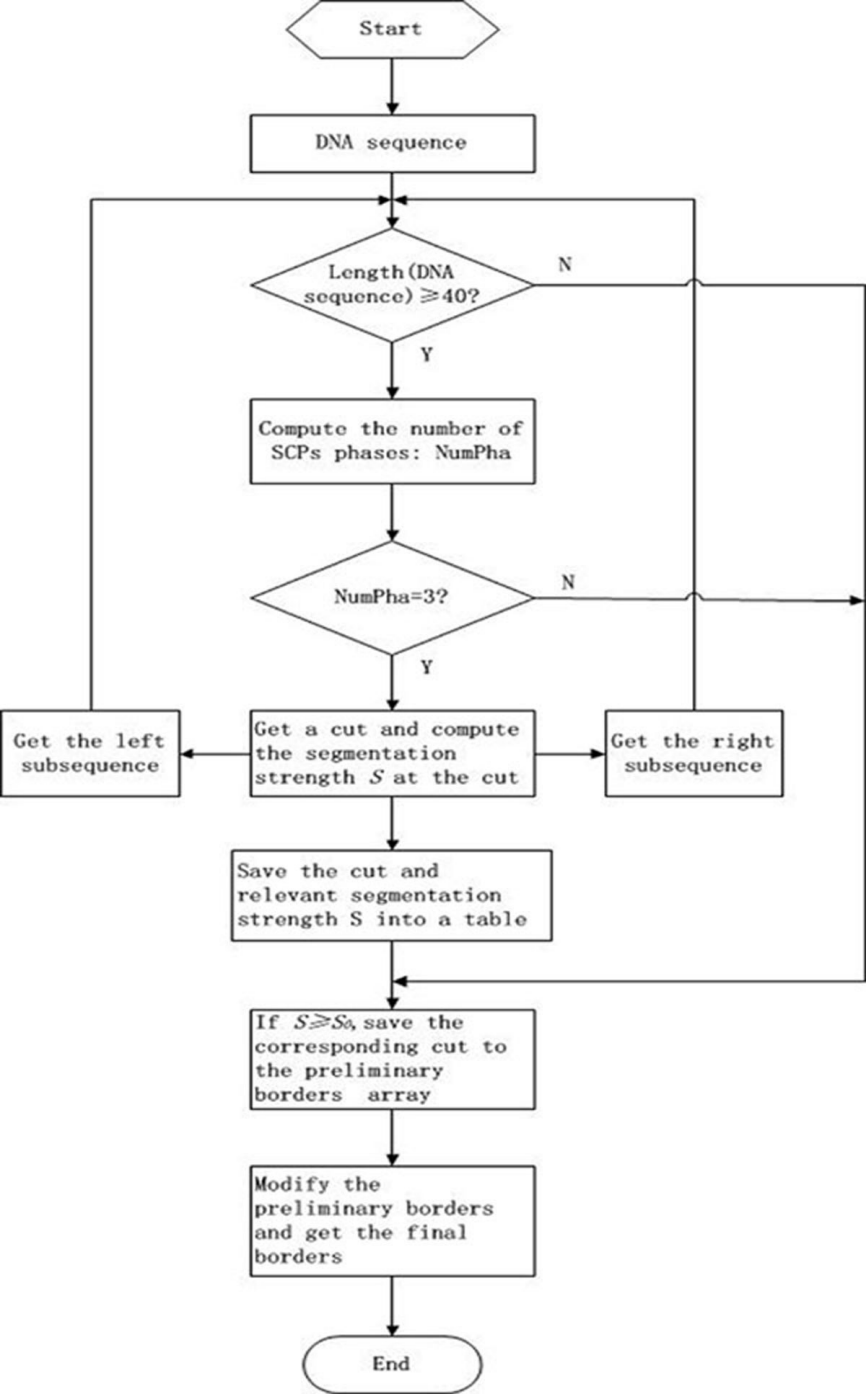
**The flow chart of the procedure for finding borders between coding and non-coding DNA regions**.

Actually, the probable borders (the significant cuts) predicted by the recursive segmentation method is generally not the actual borders but are close to them. Since it is well known that the codons at the real borders between coding and non-coding DNA regions must be one kind of start or stop codons, we could use start or stop codons like nuclear acid pattern around the border as border cut. Then, we filter the segment region less than 20 bp. The procedure for finding borders between coding and non-coding DNA regions can be described by the flow chart (Figure 1).

### Evaluation

In order to evaluate how well the predicted borders matching the actual borders between coding and non-coding regions, we use the following measure introduced by Bernaola-Galvan et al. [12].

(3) $$CBC=\frac{1}{2}\left[\underset{i}{\sum }\frac{{min}_{j}\left|b_{i}-c_{j}\right|}{N_{T}}+\underset{j}{\sum }\frac{{min}_{i}\left|b_{i}-c_{j}\right|}{N_{T}}\right]$$

Where {*b_i_*} is the set of all known borders (called KBs for short) between coding and non-coding regions, and { *c_j_*} is the set of all predicted borders (called PBs for short), and *N_T _*is the total length of the DNA sequence. The first summation measures the discrepancy between PBs and KBs by adding the distance from each KB to the closest PB, and the second summation performs the same operation, but includes the distance from each PB to the closest KB. Both are required to take into account not only the correctness for the cutting position (CBC would be zero only when the PBs overlap the KBs), but also the difference between the number of PBs and KBs. CBC can be viewed as an average of the error in determining the correct boundaries between coding and non-coding regions, so (1-CBC) is a reasonable measure of the accuracy of the method.

## Results and discussion

**Figure 2:**
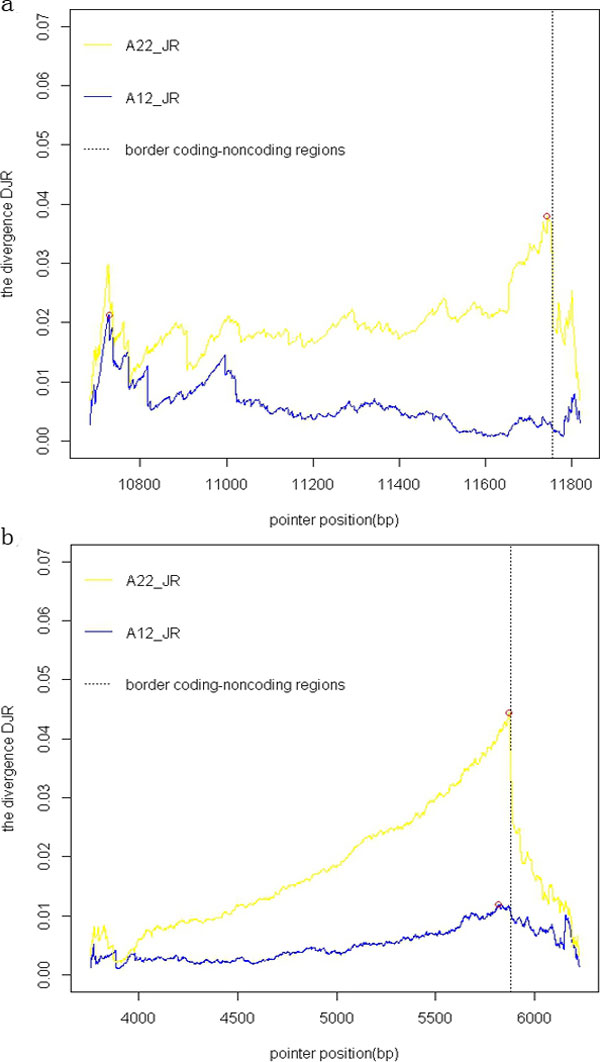
**Jensen-Rényi divergence versus cutting position for a DNA sequence**. The DNA sequence contains a coding region followed by a non-coding region. The maximum values for the divergences are circled on the graph. (a) The analyzed DNA segment was chosen from bacterium *Rickettsia prowazekii *(AJ235269, 3757-6226 bp). (b) The analyzed DNA segment was chosen from bacterium *Rickettsia prowazekii *(AJ235269, 10683-11820 bp).

In Figure 2, we plotted the Jensen-Rényi divergence (α = 0.5 and used in the following experiments as the prediction results have no change when α is adjusted from 0 to 1) with A12 [12] and A22 alphabets along a DNA segment. The DNA segment was randomly chosen from the bacterium genome *Borrelia burgdorferi *and *Rickettsia prowazekii*. In Figure 2(a), the analyzed DNA segment was chosen from bacterium *Rickettsia prowazekii *(AJ235269, 3757-6226 bp). The left part (length 2121 bp) belongs to a coding region and the right part (length 350 bp) belongs to a non-coding region. In Figure 2(b), the analyzed DNA segment was chosen from bacterium *Rickettsia prowazekii *(AJ235269, 10683-11820 bp). The left part (length 1074 bp) belongs to a coding region and the right one belongs to a non-coding region. From Figure 2, the cuts predicted by A22-JR (the method with A22 alphabet, Jensen-Rényi divergence) are closer to the real borders than those by A12-JR.

We also applied the two methods to whole genome respectively. There are multiple coding and non-coding regions in those sequences. The results are summarized in Table 3. The accuracy of A22_JR is better than that of A12_JR for each DNA sequence (*p *= 0.0015, Table 3).

**Table 3:** The maximum accuracy of different methods applied to different data sets.

Organism	GenBank ID	1-CBC(×100%)	
		
		A12-JR	A22-JR
*Rickettsia prowazekii*	AJ235269	62.50	63.85
			
*Borrelia burgdorfer*	NC_000948	69.18	70.57
	AE000783	70.48	73.18
			
*Escherichia coli*	NC_010468	72.26	75.44
	NC_008563	73.39	77.70
	NC_009837	71.45	75.68

**Figure 3:**
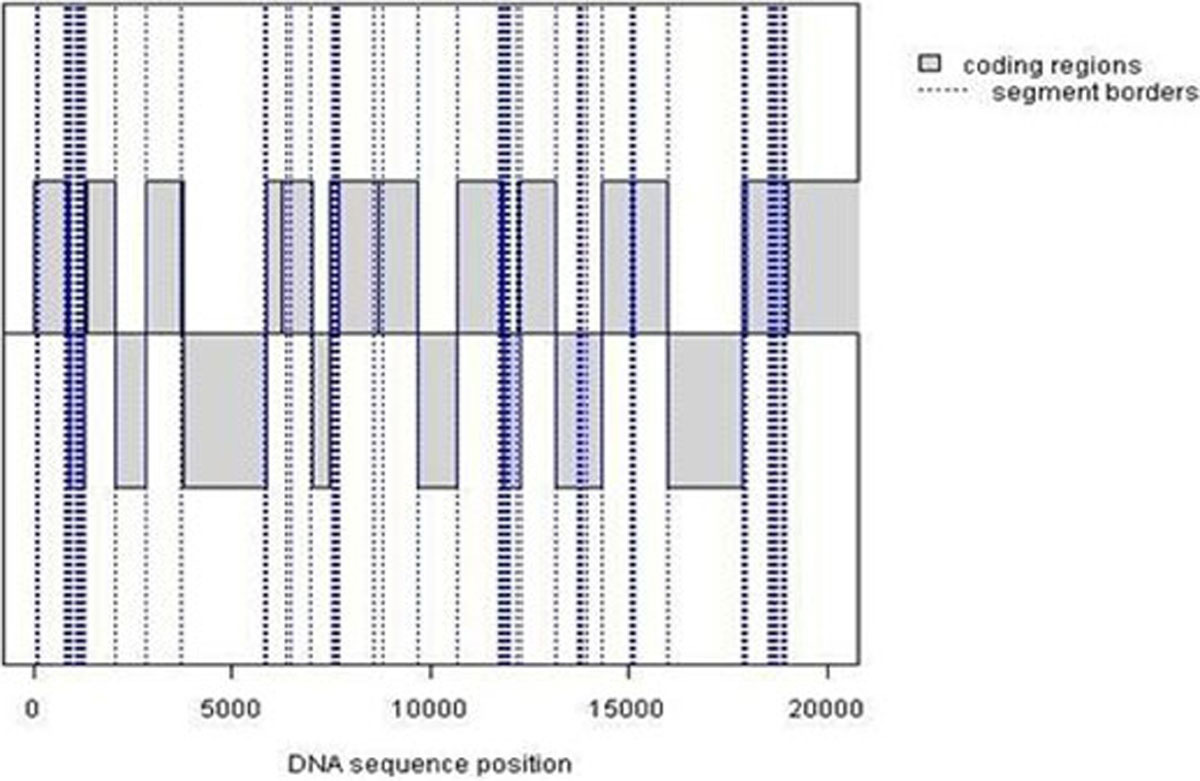
**Comparison between the known coding regions and the predicted borders of a DNA sequence**. The known coding regions are gray regions with solid lines as borders. The predicted borders (vertical dotted lines) is obtained through recursive segmentation using A22_JR (a = 0.5). The DNA sequence is from bacteria Rickettsia prowazekii and the borders. The coding regions shown downwards are on the opposite DNA strand.

For visualizing the borders predicted by our proposed method, we plotted the known coding regions in the first 22000 bp of the bacterium genome *Borrelia burgdorferi *(AE000783) and the unmodified predicted borders from our results (Figure 3).

**Figure 4:**
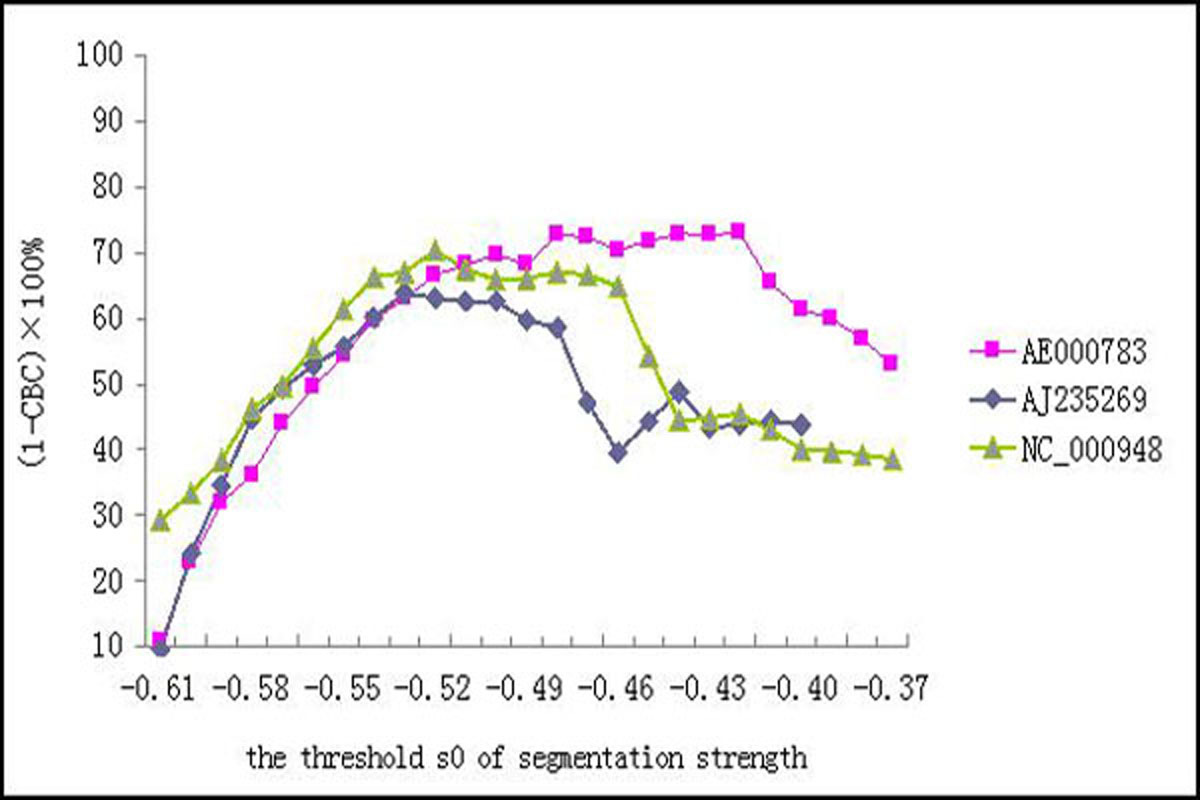
**Accuracies of recursive segmentation for different thresholds of segmentation strength**. The DNA sequences are from the genomes of *Rickettsia prowazekiiand *and *Borrelia burgdorferi *(the first 30000 bp).

Finally, we described how to choose an appropriate threshold *s*_0 _of segmentation strength. After having gotten the cuts and their corresponding segmentation strength, *s*_0 _ranged from 0.30 to 1.00 stepping by 0.01. For each *s*_0_, the accuracy was computed. From Figure 4, we can find that the accuracy is much higher when *s*_0 _is about -0.50. Thus the appropriate threshold *s*_0 _of segmentation strength can be set as -0.50.

In this study, we introduced a new segmentation method for finding the borders between coding and non-coding regions. It is based on the Jensen-Rényi divergence, a 22-symbol alphabet, and a new stopping criterion. Tested on three bacteria genomes, our method improved the accuracies of the borders detection over the previously reported A12-JR segmentation approach. Most of the existing segmentation algorithms [10,12,13] rely heavily on statistical properties of the coding, non-coding or other interested regions in DNA sequences. Moreover, since the gene-finding systems [24,26,27] use biological knowledge regarding functional sites, together with statistics for finding genes, they require extensive training on known datasets. The recursive segmentation needs no prior training. It should be noted that the value of the segmentation strength threshold *s*_0 _is generally set as -0.50 for bacterium and may be adjusted accordingly in different species. For a new unknown genomic sequence, the optimal threshold *s*_0 _of segmentation strength or significance level can be computed using the genomic sequence of the same or the closest organism.

## Conclusions

The borders between coding and non-coding regions are found more efficient and accurate will raise the vital effect for DNA sequences annotation. This paper presents a new segmentation method based on Jensen-Rényi divergence with a 22-symbol alphabet, new stopping criterion for finding the borders between coding and non-coding DNA regions in prokaryotes. For three bacteria genomes, comparing to A12_JR method, our method raised the accuracy of finding the borders between coding and non-coding regions in DNA sequences. The success comes from the utilization of the di-nucleotides and SCPs statistics in all three phases along the DNA sequence, and use of Jensen-Rényi divergence.

## Competing interests

The authors declare that they have no competing interests.
